# Mechanisms and clinical significance of Tumarkin-like phenomenon during the final step of the Epley and Semont maneuver: insights from virtual simulation and literature review

**DOI:** 10.3389/fneur.2025.1547798

**Published:** 2025-03-06

**Authors:** Ruihu Yang, Xiaokai Yang

**Affiliations:** ^1^Postgraduate Training Base Alliance of Wenzhou Medical University, Wenzhou People's Hospital, Wenzhou, Zhejiang, China; ^2^Wenzhou Third Clinical Institute Affiliated to Wenzhou Medical University, Wenzhou People's Hospital, Wenzhou, Zhejiang, China

**Keywords:** BPPV, Epley maneuver, Semont maneuver, virtual simulation, otoconia, semicircular canals, postural instability, dizziness

## Abstract

**Objectives:**

This study aims to investigate the mechanisms underlying the Tumarkin-like phenomenon during the final step of the Epley and Semont maneuvers for benign paroxysmal positional vertigo (BPPV) through virtual simulation and a comprehensive literature review. We also provide clinical recommendations to improve treatment outcomes and optimize repositioning protocols.

**Methods:**

A three-dimensional virtual simulation model was developed to accurately represent the semicircular canals, otoliths, and associated vestibular structures. Key parameters governing otolith movement were defined based on physiological data. Virtual experiments were conducted to simulate the final steps of the Epley and Semont maneuvers, allowing detailed observation of otolith movement. The study followed ethical guidelines throughout.

**Results:**

Virtual simulations revealed distinct otolith movement patterns during the Epley and Semont maneuvers. In the standard Epley maneuver, otoliths should enter the utricle before the final sitting up step, resulting in no further movement or symptoms. Conversely, in the Semont maneuver, otoliths may enter the utricle through the common crus when sitting up, potentially causing vertigo, nystagmus, and unsteadiness. Improper execution of either maneuver can lead to unexpected otolith movements and symptoms. The clinical significance of symptoms during the final step varies between the two maneuvers and is closely linked to proper execution. The study also highlights the importance of head positioning during the maneuvers, with specific angles influencing otolith movement and symptom manifestation.

**Conclusions:**

The findings provide a detailed understanding of otolith movement dynamics during the final steps of the Epley and Semont maneuvers. The results challenge existing views on the correlation between dizziness and successful repositioning, emphasizing the need for personalized treatment approaches and accurate maneuver execution. This study contributes to refining repositioning protocols, optimizing outcomes, and advancing our comprehension of BPPV dynamics. Future clinical studies are needed to verify these simulation results and develop more precise and personalized BPPV diagnosis and treatment methods.

## 1 Introduction

Benign paroxysmal positional vertigo (BPPV) is a prevalent vestibular disorder, affecting ~2.4% of the general population (1). It is characterized by brief, intense episodes of vertigo triggered by specific head movements. These episodes occur due to dislodged otoconia (small calcium carbonate crystals) that migrate into the semicircular canals, most commonly the posterior canal. The semicircular canals are part of the vestibular system and play a crucial role in maintaining balance. When otoconia enter these canals, they disrupt normal fluid dynamics and lead to inappropriate stimulation of the canal's sensory receptors during head movements, resulting in vertigo (2).

BPPV significantly affects the quality of life, particularly in older adults, due to the frequent and disabling vertigo episodes it causes, which can lead to physical limitations, psychological impact, reduced mobility, and a higher risk of falls.

The Epley and Semont maneuvers are well-established and effective interventions for repositioning displaced otoconia within the semicircular canals and alleviating symptoms of BPPV (2, 3). These maneuvers involve a series of head and body movements designed to guide the dislodged otoconia back to the utricle, where they can be absorbed and no longer cause vertigo. However, a significant characteristic of the Epley and Semont maneuvers is their potential to elicit a Tumarkin-like phenomenon.

This phenomenon is clinically defined as a sudden, self-reported sensation of being thrown to the ground, accompanied by dizziness and postural instability during the final step of the maneuver. Specifically, it occurs when patients transition from a supine position to an upright posture (4–14).

This study aims to examine the mechanisms and clinical significance of the Tumarkin-like phenomenon, particularly during the seated transition phase of the Epley and Semont maneuvers. By analyzing and reconciling conflicting research findings on the occurrence and implications of these symptoms (4), we seek to provide a deeper understanding of the Epley and Semont maneuvers' application in treating BPPV. Elucidating the underlying mechanisms may help optimize the maneuvers' efficacy, improve patient comfort, and inform clinical decision-making.

Seminal works provide a foundational understanding of the association between dizziness during the seated transition of the Semont maneuver and the success of otoconia repositioning (11–14). These studies collectively suggest that the manifestation of dizziness is not merely a side effect but a positive prognostic indicator for successful repositioning. This understanding is based on the fluid dynamics involved, where the movement of dislodged otoconia stimulates the crista ampullae as they traverse the common crus during the final step of the Semont maneuver, thus leading to dizziness (14). The proposed mechanism is that the dizziness occurs when the otoconia are successfully migrating through the semicircular canals toward the utricle (11). This consistent association has led to dizziness being considered an essential clinical sign during the Semont maneuver, guiding clinicians in assessing the effectiveness of the repositioning process (13).

In contrast, literature on the Epley maneuver presents a more complex narrative regarding the relationship between dizziness and successful repositioning. Early studies by Power et al. (6), Uneri (7), Kim (8), and Maranhão (15) indicated a consistent correlation between dizziness during the seated transition of the Epley maneuver and successful repositioning, similar to observations made with the Semont maneuver. These early findings, based on the assumption that dizziness is a sign of successful otoconia migration, laid the groundwork for interpreting dizziness during the Epley maneuver as an indicator of successful treatment and influenced treatment guidelines, leading to its widespread adoption in managing BPPV (6).

However, more recent research has questioned these established interpretations. de Morais et al. (4) and Shigeno (5) challenge the conventional understanding of the Epley maneuver's effects. Pimente et al.'s (4) recent prospective study introduces complexity by suggesting that dizziness during the final step of the Epley maneuver is not an indicator of successful otoconia repositioning, but rather a sign of an unsuccessful attempt. Their findings, based on a thorough examination of nystagmus in the fourth position of the maneuver, indicate that the presence of nystagmus is associated with unsuccessful repositioning (4). The authors propose that the nystagmus observed in the fourth position may be a result of otoconia falling back into the semicircular canal after an unsuccessful repositioning attempt. Similarly, Shigeno's study (5) highlights adverse effects of the Epley maneuver, such as anterior canal crisis, and calls for caution in assuming a positive correlation between dizziness and successful repositioning. Shigeno's (5) findings suggest that dizziness during the Epley maneuver may indicate complications rather than successful treatment, urging a reevaluation of the established interpretation.

Given these conflicting perspectives, it is crucial to reconcile the divergent findings from Pimente et al. (4) and Shigeno (5) with earlier studies (6–8). This study seeks to offer a nuanced understanding of dizziness during the Epley and Semont maneuvers by exploring the underlying mechanisms and clinical implications. By providing a more comprehensive approach to the interpretation of these symptoms, we aim to contribute to the optimization of BPPV treatment strategies, ultimately improving patient care and outcomes.

## 2 Methods

### 2.1 Virtual simulation techniques

#### 2.1.1 Calibration of membranous labyrinth model in three-dimensional space

The calibration of the membranous labyrinth model in three-dimensional space is crucial for virtual simulation. However, due to the extremely small structure of the membranous labyrinth, clinical CT/MRI examinations cannot fully display the membranous labyrinth structure. This study's membranous labyrinth model is based on the micro-CT segmented membranous labyrinth model by David et al. (16). The model was constructed based on high-resolution micro-CT scans of human temporal bones, with a spatial resolution of 13.57 μm, ensuring anatomical accuracy and precise representation of the membranous labyrinth, including the utricle, ampulla, cupula, and all three semicircular canals (16). Our innovative work involves calibrating the bony labyrinth model provided by David's team with our established standard spatial coordinate system bony labyrinth model, allowing the membranous labyrinth model to rotate accordingly and thus establishing the spatial orientation of the membranous labyrinth (17).

#### 2.1.2 Virtual simulation engine

There are various virtual simulation models to choose from. Initially, we used Blender (version 2.79b) software with the Bullet physics engine, which has the disadvantage of being a single-machine version requiring manual programming to control the movement of the semicircular canals (18). To facilitate research, we have adopted Unity 3D software (version 2020.3) and employed the NVIDIA PhysX physics engine for realistic physical simulations. The model was designed with a browser-server architecture, which is advantageous for remote access, multi-user access, supports the import and export of the membranous labyrinth model, allows for the setting of otoconia with mouse clicks, supports the setting of treatment maneuvers with quaternions/Euler angles, displays the movement speed of otoconia, and supports the export of simulation videos.

#### 2.1.3 Virtual simulation parameter settings

Setting the parameters for BPPV virtual simulation is critical. The membranous labyrinth model is set as a rigid structure, with the size and density of otoconia and the density of endolymph referencing previous research data. The resistance parameters and friction coefficient were adjusted to make the otoconia settlement speed close to 0.2 mm/s, which aligns well with clinical experience. Key Parameters:

- Otolith radius: the radius of otoconia within the model ranged from 0.5 to 15 μm, with an average radius of 7.5 μm, representing a realistic variation in otoconia size based on empirical data.
- Otolith density: set at 2.71 grams per cubic centimeter (g/cm^3^), corresponding to the typical density of calcium carbonate crystals found in the human ear.
- Endolymph density: fixed at 1 g/cm^3^, consistent with the physiological properties of the inner ear fluid.
- Buoyancy-induced acceleration: set at 3.62 m per second squared (m/s^2^), this parameter accounts for the differential density between otoconia and endolymph, reflecting the net acceleration experienced by the otoconia due to the combined effects of gravity, buoyancy, and drag forces under normal conditions (17, 19– 22).

### 2.2 Position of otolith settlement in sitting position

In the upright seated position, otoliths were observed to settle in the long arm of the left posterior semicircular canal, consistent with left posterior canal BPPV. Setting otoliths in other appropriate areas as needed.

### 2.3 Experimental design

Virtual experiments were meticulously designed to simulate the Epley and Semont maneuvers, with a specific focus on the final steps that involve transitioning from a supine to an upright position. These experiments aimed to provide critical insights into the movement of otoliths and the forces exerted on them during these maneuvers.

#### 2.3.1 Semont maneuver simulation

1. Initial position: the virtual patient was seated with the head turned 45 degrees away from the affected ear.
2. Lateral decubitus position: the patient was rapidly moved to the lateral decubitus position on the affected side, with the head turned 45 degrees upward. The patient's nose should be pointing slightly upward. This position was maintained for 1 min.
3. Opposite lateral decubitus: after maintaining this position for about 1 min, the patient was swiftly moved to the opposite lateral decubitus position, without changing the head orientation relative to the body. In this position, the patient's nose should be pointing downward. This position was maintained for 1 min.
4. Seated position: the patient was then brought back to the seated position.
5. For non-standard Semont maneuver, on Lateral Decubitus Position, the head is in the horizontal plane to 20° below the horizontal plane. On Opposite Lateral Decubitus, the head is in the horizontal plane to 20° below the horizontal plane (12, 23).

#### 2.3.2 Epley maneuver simulation

1. Initial position: the virtual patient was positioned in a seated position on the examination table, with the head turned 45 degrees toward the affected ear.
2. Supine position: the patient was then moved to a supine position, maintaining the 45-degree head turn toward the affected ear. The patient's head was extended about 30 degrees over the edge of the table. This position was maintained for 30 s.
3. Contralateral head rotation: while maintaining the supine position, the patient's head was rotated 90 degrees to the opposite (unaffected) side. This position was maintained for 30 s.
4. Lateral decubitus: the patient was then rolled onto their side, in the direction of the unaffected ear. The patient's nose should be pointing 45 degrees downward. This position was maintained for 30 s.
5. Seated position: finally, the patient was brought back to a seated position, with the head tilted forward 45 degrees and maintained in this position for 5 min.

**Figure 1:**
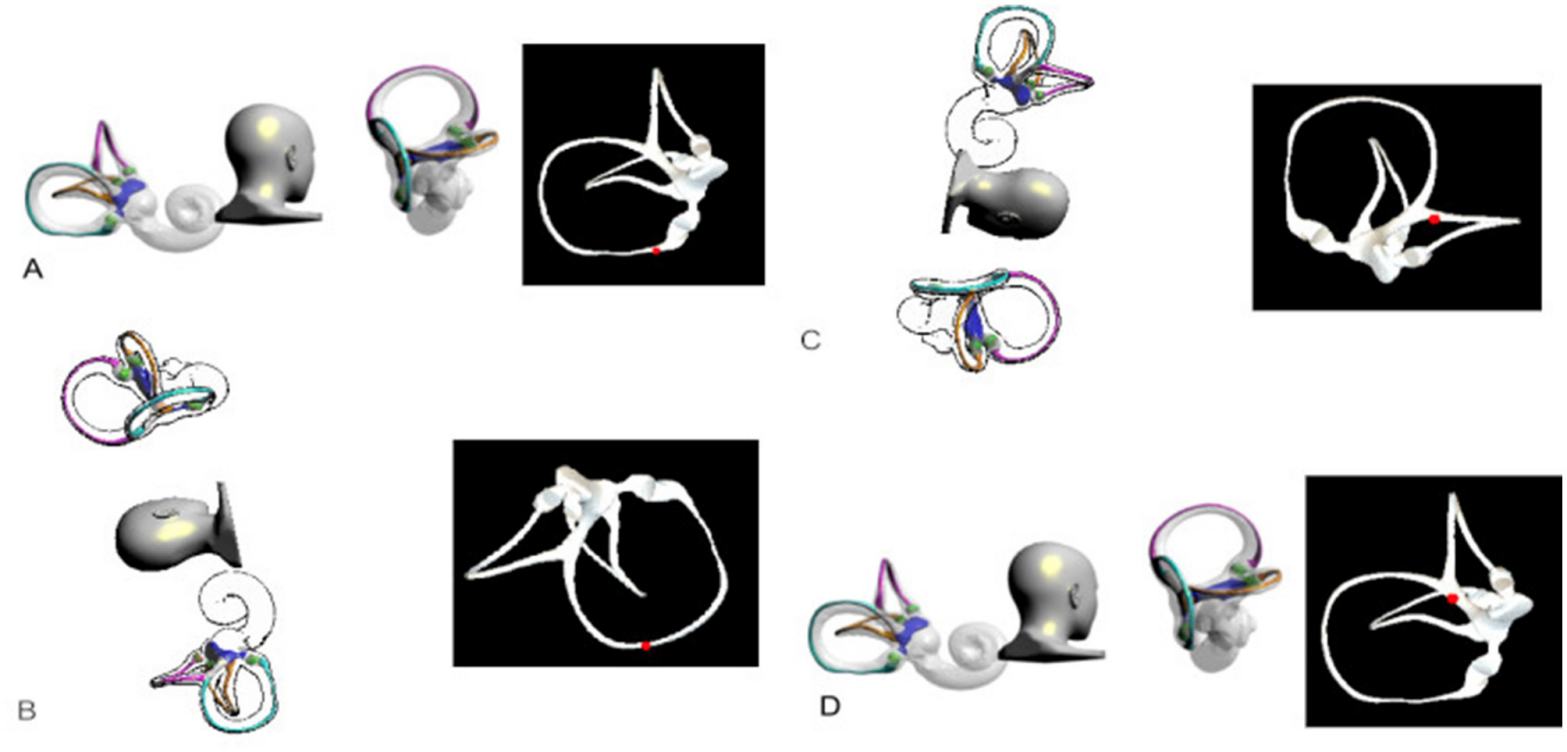
Left Semont maneuver. **(A)** Initial Position: The patient was seated with the head turned 45 degrees away from the affected ear. **(B)** Lateral Decubitus Position: The patient was rapidly moved to the lateral decubitus position on the affected side, with the head turned 45 degrees upward. The patient's nose should be pointing slightly upward. This position was maintained for 1 min. **(C)** Opposite Lateral Decubitus: After maintaining this position for about 1 min, the patient was swiftly moved to the opposite lateral decubitus position, without changing the head orientation relative to the body. In this position, the patient's nose should be pointing downward. This position was maintained for 1 min. **(D)** Seated Position: The patient was then brought back to the seated position.

**Figure 2:**
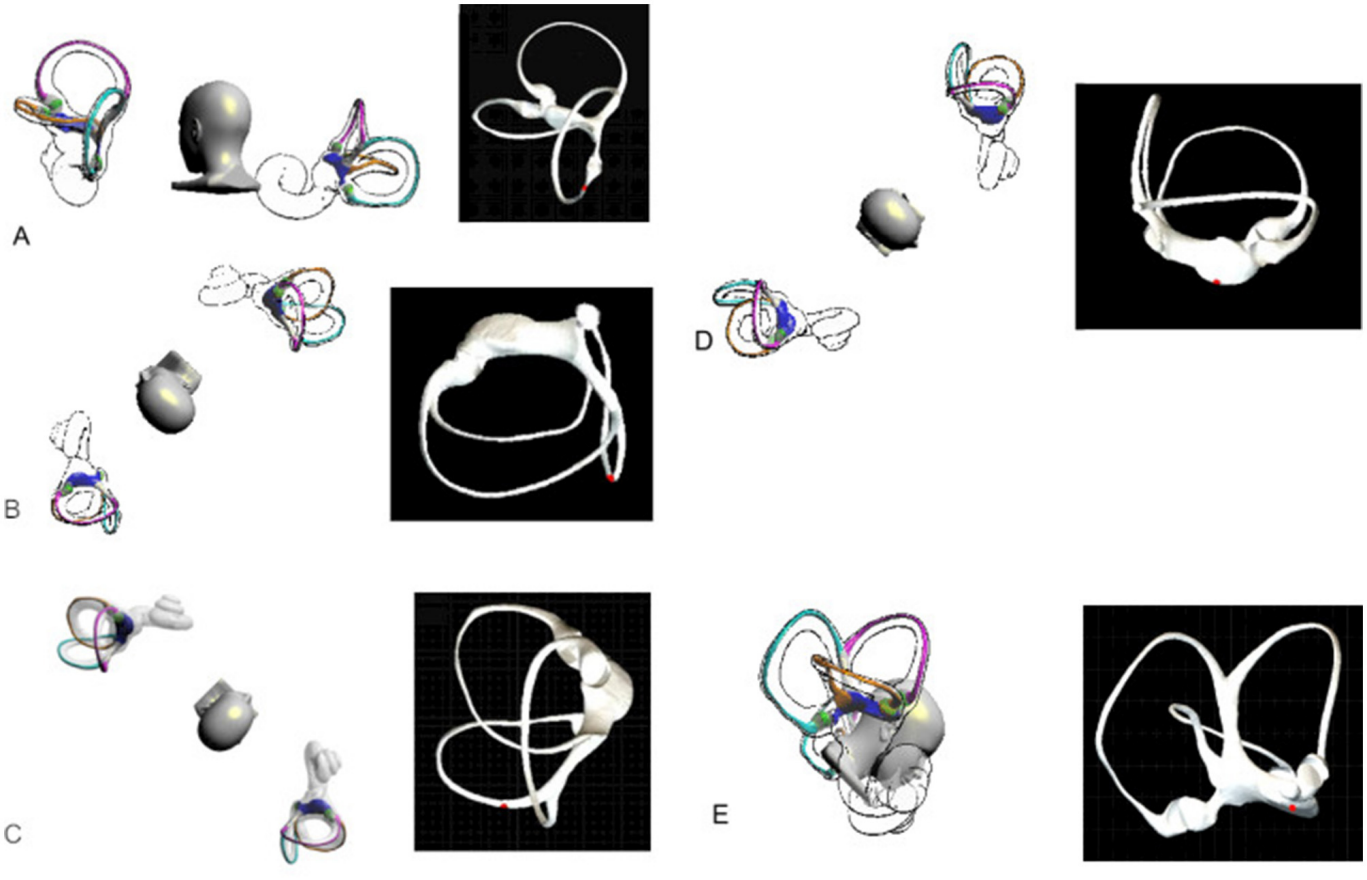
Left Epley maneuver. **(A)** Initial Position: The patient was positioned in a seated position, with the head turned 45 degrees toward the affected ear. **(B)** Supine Position: The patient was then moved to a supine position, maintaining the 45-degree head turn toward the affected ear. The patient's head was extended about 30 degrees over the edge of the table. This position was maintained for 30 s. **(C)** Contralateral Head Rotation: While maintaining the supine position, the patient's head was rotated 90 degrees to the opposite (unaffected) side. This position was maintained for 30 s. **(D)** Lateral Decubitus: The patient was then rolled onto their side, in the direction of the unaffected ear. The patient's nose should be pointing 45 degrees downward. This position was maintained for 30 s. **(E)** Seated Position: Finally, the patient was brought back to a seated position, with the head tilted forward 45 degrees and maintained in this position for 5 min.

During each maneuver, the simulation allowed for detailed observation and recording of otolith movement within the membranous labyrinth (Figures 1, 2).

For the non-standard Epley maneuver, after step C, the head was rotated from 90° to 180° from the horizontal to the healthy side position, moving through the healthy side to the prone position (5).

#### 2.3.3 Visualization

1. Advanced visualization techniques, such as 3D rendering and animation, were utilized to depict otolith movement patterns and the forces acting on them during the maneuvers.
2. These visualizations provided a clearer understanding of the complex dynamics involved in BPPV and offered insights into the effectiveness of the Epley and Semont maneuvers.

#### 2.3.4 Nystagmus pattern analysis

We analyzed the nystagmus patterns corresponding to different otolith positions and movement directions according to the Table 1.

**Table 1:** Nystagmus patterns and otolith movement.

**Otolith Position**	**Movement direction**	**Nystagmus pattern**
Long arm of posterior canal	Toward ampulla	Downbeat and counterclockwise torsional
Long arm of posterior canal	Away from ampulla	Upbeat and clockwise torsional
Long arm of Superior canal	Away from ampulla	Downbeat and clockwise torsional
Utricle	Displacement	Postural instability
Short arm of posterior canal	Toward ampulla	Upbeat and clockwise torsional

## 3 Results

Our study offers a comprehensive analysis of otolith movement during the execution of the Epley and Semont maneuvers for benign paroxysmal positional vertigo (BPPV).

### 3.1 Otolith movement during the Epley maneuver

**Figure 3:**
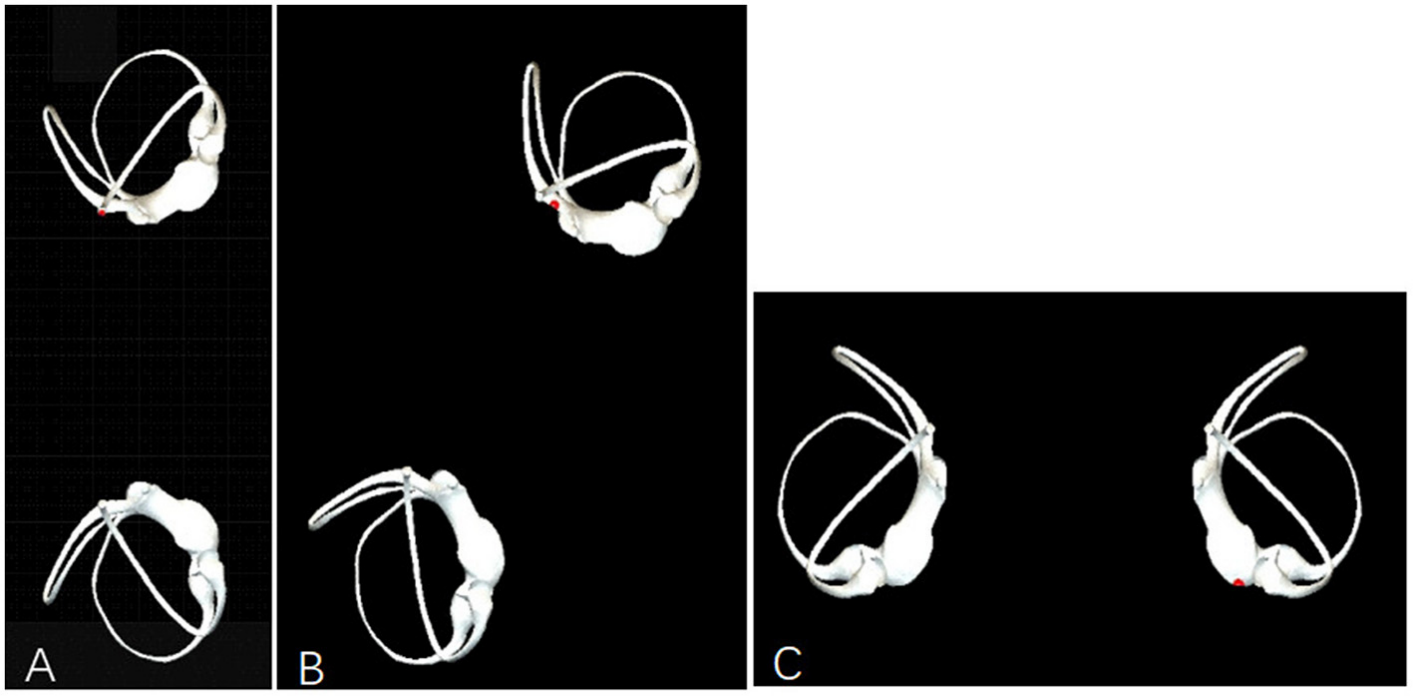
Observation of otolith movement during the non-standard left Epley maneuver. **(A)** In Step D, the patient is positioned in a healthy-side lying position at 90° from the horizontal plane, allowing the otoliths to be displaced into the superior semicircular canal. **(B)** In Step D, the patient is positioned at 120° from the horizontal plane toward the healthy side, keeping the otoliths within the common crus. **(C)** In Step D, the patient is placed in a prone position, which is 180° from the horizontal plane toward the healthy side, facilitating the otoliths' passage through the common crus into the utricle.

We conducted a detailed investigation of otolith movement during the Epley Maneuver, taking into account the potential variations in head positioning due to procedural inconsistencies or non-standardized maneuvers. Specifically, the final step of the Epley Maneuver, as recommended by guidelines, does not require a 45-degree head tilt downward (Simulations 1, 2, and 3). Simulation 1—Epley Maneuver: Observation of Otolith Movement on the Long Arm Side of the Left Posterior Semicircular Canal (Supplementary video S1). Simulation 2—Epley Maneuver: Observation of Otolith Movement on the Short Arm Side of the Left Posterior Semicircular Canal (Supplementary video S2). Simulation 3—Epley Maneuver: Observation of Otolith Movement Within the Left Utricle (Supplementary video S3). We also explored variations in head rotation angles during step D of the Epley Maneuver, ranging from 90 to 180 degrees from the supine position, through additional simulations. Simulation 4—Epley Maneuver: Step D—Lateral Decubitus Position (Supplementary video S4). Simulation 5—Epley Maneuver: Step D—Semi-Prone Position (Supplementary video S5). Simulation 6—Epley Maneuver: Step D—Prone Position (Supplementary video S6). For non-standard Epley maneuvers, if step D head rotation varied from 90° to 120° from the horizontal to the healthy side position, the otoliths remained in the common crus or were displaced to the superior semicircular canal, instead of entering the utricle. This resulted in vertigo, nystagmus, and body unsteadiness when sitting up (Figures 3A,B). Conversely, if the head rotation in step D was between 121° and 180°, the otoliths passed through the common crus into the utricle, with no further movement through the semicircular canals during sitting up, thus avoiding dizziness, nystagmus, or unsteadiness (Figure 3C). In the traditional Epley maneuver, the final step involves otoliths rolling a considerable distance from the long arm of the posterior semicircular canal into the short arm side. This movement is not directly associated with immediate dizziness and unsteadiness upon returning to the seated position but may be a significant cause of delayed postural instability. To prevent otoliths from entering the short arm side of the posterior semicircular canal in the final step, we modified the Epley maneuver by adding a 45-degree head tilt downward in step E. We first observed otolith movement on the long arm side of the left posterior semicircular canal (Simulation 7). Simulation 7—Epley Maneuver: Final Step with 45° Nose-Down Position—Observation of Otolith Movement on the Long Arm Side of the Left Posterior Semicircular Canal (Supplementary video S7). In the initial position, otoliths were stationary within the left posterior canal. Upon assuming the supine position with a 30° head extension, otoliths moved away from the ampulla, accompanied by upbeat and clockwise torsional nystagmus. A subsequent 90° head rotation to the unaffected side resulted in further otolith migration away from the ampulla, with a diminished nystagmus response. In the lateral decubitus position, otoliths continued their trajectory, ultimately entering the utricle. The final seated position showed no further otolith movement or nystagmus. We also observed otolith movement in multiple positions with the modified maneuver (Simulation 8). Simulation 8—Epley Maneuver: Final Step with 45° Nose-Down Position—Multi-Position Otolith Movement Observation in the Left Posterior Semicircular Canal (Supplementary video S8). Based on the hydrodynamics of otolith movement and principles of vestibular physiology, we analyzed the otolith movement at different steps and positions during the Epley maneuver, as well as the associated nystagmus characteristics (Table 2).

**Table 2:** Biomechanical responses of otoliths during left Epley repositioning maneuver.

**Otolith Position**	**Step A**	**Step B**	**Step C**	**Step D**	**Step E**
LPSa	–	Short cristal migration, excitatory, ↑↶	+/–, Limited displacement	+/–, ampullopetal reflux, inhibitory, ↓↷	+/–, ampullofugal escape, excitatory, ↑↶
LPLa	–	++, ampullofugal, excitatory, ↑↶	+, ampullofugal, excitatory, ↑↶	++/+, ampullofugal and enters utricle, excitatory, ↑↶	–, move in utricle
LU	–	+-/-, move in utricle and near short arm of the ASC, excitatory, ↓↶	+, enters utricle and move to superior semicircular canal, inhibitory, ↑↷	–, no movement	++/+, ampullofugal and enters utricle, excitatory, ↓↷
LP_C	–	+, ampullopetal, inhibitory,↓↷	+, ampullofugal, excitatory, ↑↶	++/+, ampullofugal and enters utricle, excitatory, ↑↶	–, move in utricle

LPSa, left posterior semicircular canal short arm side; LPLa, left posterior semicircular canal long arm side; LU, left utricle; LP_C, left posterior semicircular canal near the common crus.

Nystagmus intensity: –(No nystagmus), + (Mild), ++ (Moderate), +++ (Strong); Nystagmus Direction: → (Rightward), ← (Leftward), ↑ (Upbeat), ↓ (Downbeat), ↷ (Clockwise), ↶ (Counterclockwise), – (No direction).

### 3.2 Otolith movement during the Semont maneuver

The Semont maneuver was similarly analyzed for left posterior canal BPPV (Table 3). From the initial position, the lateral decubitus transition induced otolith movement away from the ampulla, eliciting upbeat rotatory nystagmus toward the ground.

**Table 3:** Otolith movement and nystagmus during Semont maneuver (left posterior canal BPPV).

**Maneuver step**	**Otolith movement**	**Observed nystagmus**
Initial position	Stationary in left posterior canal	None
Lateral decubitus position	Movement away from ampulla	Upbeat and clockwise torsional
Opposite lateral decubitus	Continued movement away from ampulla	Upbeat and clockwise torsional
Seated position	Movement into utricle	Upbeat and clockwise torsional

The opposite lateral decubitus position facilitated further otolith migration, with the otoliths moving into the superior semicircular canal. When sitting up, the otoliths traveled through the common crus and entered the utricle, resulting in vertigo, nystagmus, and unsteadiness of the body.

**Figure 4:**
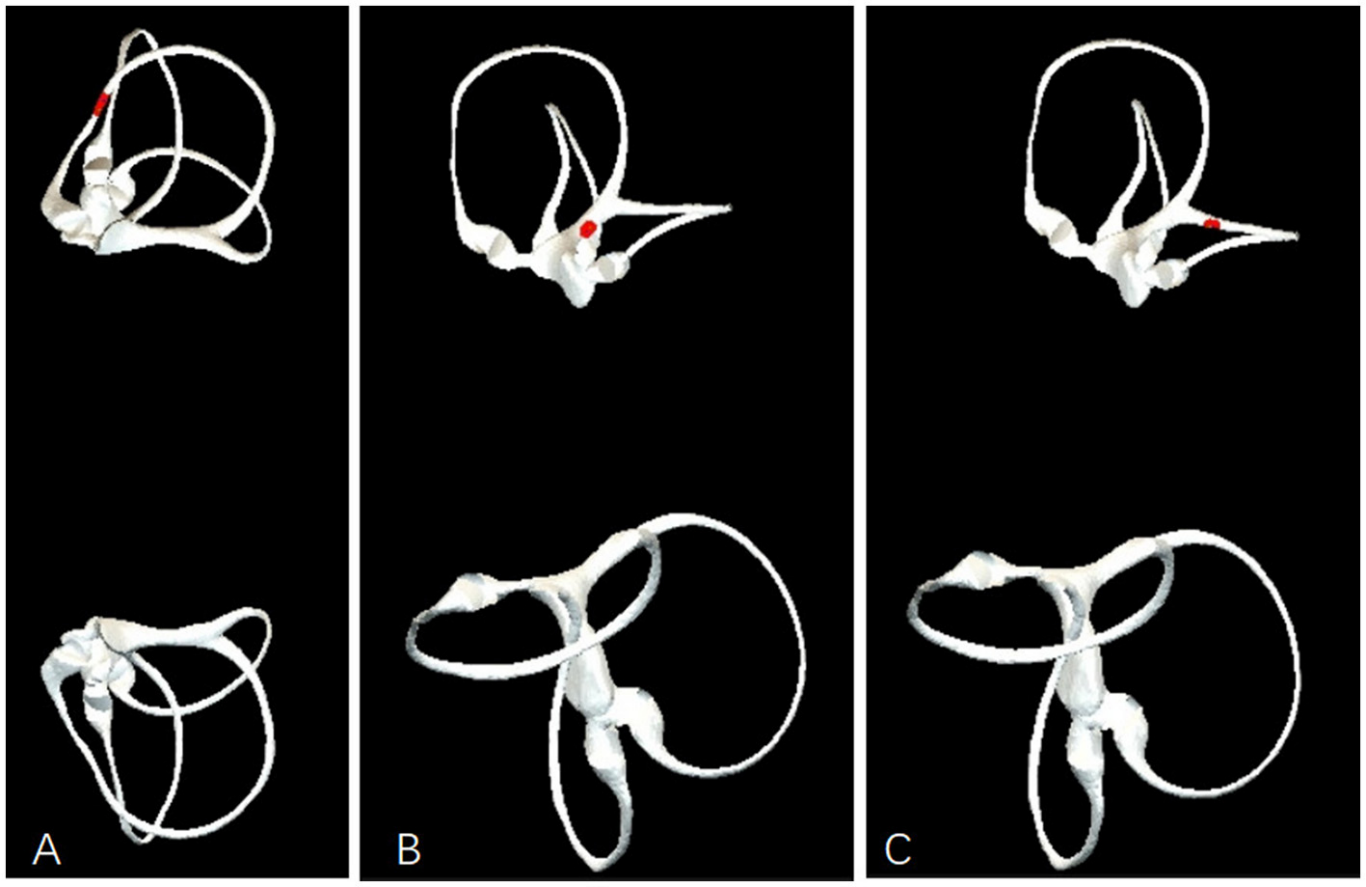
Observation of Otolith movement during the non-standard lef semont maneuver. **(A)** In the lateral decubitus position above the horizontal plane, even if the opposite lateral decubitus is below the horizontal plane by up to 20 degrees, it still cannot prevent ampullofugal movement of the otoliths. **(B)** In the lateral decubitus position below the horizontal plane, with the opposite lateral decubitus at or above the horizontal plane, the otoliths can directly enter the utricle through the common crus. **(C)** In the lateral decubitus position below the horizontal plane, with the opposite lateral decubitus also below the horizontal plane, the otoliths will migrate to the superior semicircular canal.

In the lateral decubitus position, the head should be positioned below the horizontal plane to ensure that the otoliths located at various positions along the long arm of the posterior semicircular canal can achieve sufficient ampullofugal movement. Otherwise, in the opposite lateral decubitus position, the otoliths will not move away from the ampulla, but instead toward it (Figure 4A). This would lead to a failed repositioning and no vertigo, nystagmus or unsteadiness when sitting up (24) (Simulation 9).

Simulation 9—Semont Maneuver: Step B: Lateral Decubitus Position Above the Horizontal Plane (Supplementary video S9).

When the head is maintained at or above the horizontal plane during the opposite lateral decubitus maneuver, the otoliths are able to directly enter the utricle via the common crus (Figure 4B) (Simulation 10).

Simulation 10—Semont Maneuver: Step C: Opposite Lateral Decubitus with Head at the Horizontal Plane (Supplementary video S10).

Conversely, if the head is positioned even marginally below the horizontal plane, the otoliths will instead migrate into the superior semicircular canal (Figure 4C) (Simulation 11).

Simulation 11—Semont Maneuver: Step C: Opposite Lateral Decubitus with Head Below the Horizontal Plane (Supplementary video S11).

In clinical practice, during the opposite lateral decubitus position, the head is usually positioned below the horizontal plane. This allows the otoliths to enter the utricle through the common crus when sitting up, resulting in the characteristic vertigo, nystagmus and unsteadiness.

If the head is below the horizontal plane during the lateral decubitus position and remains above the horizontal plane during the opposite lateral decubitus position, then sitting up would not provoke the typical symptoms.

When sitting up, lowering the head more than 30 degrees helps to avoid the otoliths entering the short arm side of the posterior semicircular canal.

For a more detailed visual representation of the otolith movement during the Epley and Semont maneuvers, refer to Animations in the Appendix. Those animations illustrate the dynamic process of otolith migration within the semicircular canals, complementing the biomechanical analysis presented in this section.

### 3.3 Comparative analysis of maneuvers

Both the Epley and Semont maneuvers demonstrated efficacy in repositioning otoliths from the affected posterior semicircular canal to the utricle. The Epley maneuver involved a more gradual repositioning process through multiple steps, while the Semont maneuver utilized more abrupt positional changes. Nystagmus patterns correlated closely with otolith movement in both maneuvers, with the most pronounced nystagmus observed during initial repositioning movements. In the standard Epley maneuver, when sitting up in the final step, there should be no vertigo, nystagmus, or unsteadiness. However, in the standard Semont maneuver, when sitting up in the final step, there may be vertigo, nystagmus, and unsteadiness. In an improper execution of either the Epley or Semont maneuver, sitting up in the final step may result in the opposite of the expected symptoms—i.e., the Epley maneuver may result in vertigo, nystagmus and unsteadiness, while the Semont maneuver may not. Therefore, the clinical significance of experiencing vertigo, nystagmus, and unsteadiness when sitting up after a repositioning maneuver is not only related to the choice of technique (Epley vs. Semont), but also closely linked to the proper execution of the specific maneuver.

## 4 Discussion

The Tumarkin-like phenomenon observed during the final step of the Epley and Semont maneuvers has significant clinical implications. Our virtual simulation results and literature review provide insights into the potential mechanisms behind this phenomenon. The clinical manifestations of the Tumarkin-like phenomenon, including sudden onset of dizziness, postural instability, and a sensation of falling, are critical for understanding its impact on patient outcomes. In the context of the Epley maneuver, the occurrence of the Tumarkin-like phenomenon may indicate improper execution of the maneuver, leading to unsuccessful repositioning of the otoconia. Conversely, in the Semont maneuver, the presence of these symptoms may suggest successful repositioning, as the otoconia move through the common crus into the utricle. This distinction highlights the importance of accurate maneuver execution and the need for clinicians to be aware of the specific characteristics of each technique.

### 4.1 Differences in clinical significance of symptoms in the final step of Epley and Semont maneuvers

Virtual simulation results show that although the Epley and Semont maneuvers are based on similar repositioning principles, there are significant differences in otolith movement patterns during the final step, leading to different clinical implications:

#### 4.1.1 Epley maneuver

The otolith movement pattern and its clinical significance in the final step of the Epley maneuver are complex:

1. The traditional view suggests that the occurrence of nystagmus and body instability in the final step may indicate reverse otolith movement toward the ampulla, associated with unsuccessful repositioning. This interpretation is similar to the mechanism of reversal nystagmus when sitting up during the Dix-Hallpike test.
2. However, Oh et al. proposed a different perspective. They found that the mechanism of nystagmus when sitting up during the Epley maneuver might differ from that of the Dix-Hallpike test:
  (a) Reversal nystagmus when sitting up during the Dix-Hallpike test is usually interpreted as otolith movement toward the ampulla, indicating persistent BPPV.
  (b) In contrast, Oh et al. (10) observed that many patients showed effective treatment after a single Epley maneuver, even with nystagmus when sitting up.
  (c) However, recent research by Pimentel et al. found that patients with downbeating and torsional nystagmus toward the opposite direction of the diagnostic nystagmus in the fourth position of the Epley maneuver all required a second maneuver. Patients without nystagmus in this position all showed resolution after the first Epley maneuver, supporting the traditional view (4).
3. Our virtual simulation shows that during the contralateral supine position of the Epley maneuver, otoliths from the posterior semicircular canal should have already entered the utricle through the common crus. Therefore, there should be no otolith movement within the semicircular canal when sitting up. This also supports the traditional view.

#### 4.1.2 Semont maneuver

1. If performed correctly, the occurrence of nystagmus and body instability in the final step may indicate that otoliths have entered the utricle through the common crus, suggesting successful repositioning. This is consistent with the findings of Maranhão et al. (15), who observed good treatment outcomes in patients experiencing the "Tumarkin-like phenomenon."
2. However, our simulation results suggest that if the Semont maneuver is not performed correctly, there may be no otolith movement within the semicircular canal when sitting up. Therefore, if nystagmus and body instability occur, it might indicate unsuccessful repositioning.
3. The *in vitro* study by Obrist et al. (25) provided important insights into the determinants for a successful Sémont maneuver (SM). A critical factor identified was the angle of the body movements during the maneuver. The researchers found that without extending the movements beyond the horizontal plane, the SM was not successful in their semicircular canal model (25). However, when the movements were extended by at least 20° below the horizontal line, referred to as the "Sémont+" technique, the success rate of the SM was significantly improved (25).

### 4.2 Mechanisms of the Tumarkin-like phenomenon

Indeed, there is currently a lack of a unified terminology to describe the phenomenon of dizziness, nystagmus, and instability that occurs during the final step of the BPPV repositioning maneuver, from the supine to the sitting position. This phenomenon has been variously referred to as panic, Tumarkin-like phenomenon, trunk retropulsion, postural control loss, or anterior canal crisis (5–8, 15).

We have adopted the term "Tumarkin-like phenomenon" in our study because it is well-defined and has been widely recognized in the field. Notably, Kim et al. (26) recently used the term "postural crisis" and conducted a meta-analysis of the relevant literature.

It is important to clarify that "sitting-up vertigo" and "type 2 BPPV" are not specific to the symptoms that appear during the final step of the BPPV repositioning maneuver. Instead, they refer to broader concepts that are not directly comparable to the phenomenon we are discussing.

Literature proposes several possible mechanisms to explain the Tumarkin-like phenomenon observed during BPPV treatment:

1. Otoliths entering the utricle: This phenomenon may be related to otoliths entering the utricle, similar to Tumarkin's otolithic crisis in Meniere's disease patients (7, 15).
2. Otoliths migrating to the anterior semicircular canal: This could be due to incorrect execution of the Epley maneuver, causing otoliths to move into the anterior semicircular canal and then move away from the ampulla when sitting up (5).
3. New position of otoliths in the vestibule: This phenomenon might be due to otolith redistribution in the vestibule (10).
4. Otoliths moving toward the ampulla: For some reason, otoliths in the semicircular canal may fail to reposition into the utricle, leading to movement toward the ampulla when sitting up (4). This stimulus causes the down-beating and torsional nystagmus toward the opposite side of the diagnostic nystagmus (4).
5. Controversy over otolith movement within the utricle: There is ongoing debate regarding whether the movement of otoliths within the utricle can induce nystagmus. Clinical observations suggest that dizziness and nystagmus during the final step of the Epley maneuver typically indicate unsuccessful repositioning. Mathematical models also indicate that otolith movement within the utricle does not generate significant hydrodynamic forces, making it unlikely to explain the Tumarkin-like phenomenon.
6. Otoconia entering the posterior short arm may be a plausible cause of the Tumarkin effect. For example, the prolonged dizziness and unsteadiness after sitting up are difficult to explain solely by the reverse movement of otoconia, which should occur immediately upon sitting up. Simulation experiments have shown that without the nose-down tilt in the final step of the Epley maneuver, otoconia from the utricle can slowly roll into the short arm of the posterior canal, potentially causing hydrodynamic effects (22, 27).

Our clinical experience shows that dizziness, nystagmus, and body instability during the final step of sitting up in the Epley maneuver are always associated with unsuccessful repositioning. This is consistent with Pimentel et al.'s report and our virtual simulation results. If the Epley maneuver is not performed correctly, it is indeed possible for otoliths from the posterior semicircular canal to enter the anterior semicircular canal via the common crus during the contralateral supine position, and then enter the utricle when sitting up. This might be a mechanism for some patients who experience dizziness and nystagmus in the final step but still show effective treatment. The reported incidence of this phenomenon varies from 6 to 15% in different studies (5, 7, 10, 15). These differences may reflect variations in operational techniques, observation methods, and definitions of the "Tumarkin-like phenomenon". The characteristics of nystagmus when sitting up are crucial for clarifying the mechanism. If the nystagmus is consistent with the reverse nystagmus induced by the Dix-Hallpike test, it should be considered that otoliths have moved in the opposite direction, and the treatment should be deemed unsuccessful. Conversely, if the nystagmus characteristics are consistent with those induced by the Dix-Hallpike test, it suggests that the otoliths are still moving in the same direction, and the treatment should be considered successful. However, only a few studies have clearly recorded and analyzed the characteristics of nystagmus induced when sitting up during the Epley maneuver repositioning process (4). This lack of comprehensive data represents a significant gap in our understanding of the "Tumarkin-like phenomenon" and its implications for BPPV treatment outcomes.

### 4.3 Final upright position and associated risks

The Epley maneuver concludes with the patient in a 45° nose-down position (Figure 2E), whereas the Semont maneuver terminates in a fully upright posture (Figure 1D). While patients must ultimately resume daily activities in an upright position, this transition introduces a theoretical risk of free-floating otoconia entering the short arm of the posterior semicircular canal (PSC). This mechanism has been proposed in prior computational studies as a potential contributor to the Tumarkin-like phenomenon. However, it is critical to distinguish that the Tumarkin-like phenomenon typically manifests during the final transition from supine to seated positions in repositioning maneuvers. For the Epley maneuver, our simulations confirm that otoconia do not migrate into the short arm of the PSC during this critical step, suggesting that alternative mechanisms underpin this phenomenon.

### 4.4 Clinical strategies for risk mitigation

To mitigate post-repositioning complications, we follow a comprehensive set of protocols that include maintaining a 45° nose-down position during the Epley Maneuver, which is essential for directing otoconia away from the posterior semicircular canal's (PSC) short arm during the final transition to a seated position, particularly when patients have difficulty maintaining adequate head flexion. Additionally, we emphasize the prolonged maintenance of the final position, holding the 45° nose-down position for at least 5 min to enhance otoconial adherence to the utricular macula (28), thus reducing the likelihood of recanalization. Following the maneuver, we conduct immediate positional tests such as the Dix-Hallpike to confirm symptom resolution and perform a standardized bow-and-yaw maneuver (29) to dislodge any residual otoconia from the PSC's short arm. Patients are also advised to perform the bow-and-yaw maneuver before going to bed to clear any otoconia that may have entered the short arm of the PSC, and we recommend sleeping in a lateral decubitus position to avoid supine positions that could allow otoconia from the utricle to re-enter the semicircular canals.

### 4.5 Technical limitations on cupulolithiasis

Although we acknowledge the clinical significance of cupulolithiasis, its explicit simulation was not included in this study due to the following reasons: Pathophysiological Focus: Our study primarily investigates the mechanisms underlying the Umarkin-like Phenomenon, which involves free-floating otoconial reflux. In contrast, cupulolithiasis represents a distinct diagnostic entity characterized by adhesive interactions between particles and the cupula. The pathophysiological mechanisms of these two conditions are fundamentally different, and thus, our focus on free-floating otoliths does not directly address cupulolithiasis. Modeling Constraints: Our current virtual simulation capabilities are limited to analyzing the movement of mobile otoliths under gravity-driven motion. The complex mechanics of cupular adhesion, which involve intricate particle-surface interactions, would require advanced algorithms and computational models that are beyond our current technical capabilities. Therefore, we were unable to incorporate cupulolithiasis into our simulation framework. However, it can be analyzed indirectly through the spatial attitude of the crista.

### 4.6 Clinical recommendations

Based on virtual simulation results and literature review, we propose the following clinical recommendations:

1. After performing the Epley or Semont maneuver, patients should be supported for at least 1 min to prevent potential falls. This is consistent with Uneri's recommendation (7).
2. For the Epley maneuver carried out in strict accordance with the specifications, if dizziness, nystagmus, or body instability occurs when sitting up, it strongly suggests treatment failure. It is recommended to re-check with the Dix-Hallpike test and repeat the Epley maneuver. The occurrence of the Tumarkin-like phenomenon often suggests that multiple repositioning attempts may be necessary. In such cases, clinicians should consider modifying the repositioning technique, such as extending the duration of the contralateral supine position or ensuring proper head positioning. Educate patients and their families about the possibility of the Tumarkin-like phenomenon and its implications. This can help manage expectations and reduce anxiety, especially among the elderly and those sensitive to vertigo.
3. When performing the Epley maneuver, special attention should be paid to the duration of the contralateral supine position and the angle of head bowing when sitting up. Maintaining the contralateral supine position for 5 min or until dizziness and nystagmus occur can help improve repositioning efficiency and reduce the chance of reverse otolith movement when sitting up (30).

Shigeno recommends preventing head rotation beyond 135° toward the healthy side and avoiding the head tilting downwards (5). However, the results of our simulation study showed that the head should not be turned downward in order to avoid otolith displacement into the anterior semicircular canal, but turning the head more than 135 degrees does not result in otoliths entering the superior semicircular canal. On the contrary, turning the head <120 degrees, the otoliths will stay at the common crus or enter the superior semicircular canal, and thus, in the sitting up is the otoliths enter the utricle via the common crus.

### 4.7 Study limitations and future directions

While virtual simulation provides valuable insights, it also has some limitations:

1. Model simplification: our virtual model inevitably simplifies the complex structures and physiological processes of the inner ear.
2. Individual differences: virtual simulation cannot fully reflect the individual differences between patients.
3. The limitations of the Unity 3D and PhysX engine should be clearly stated: While these tools are suitable for real-time simulations, they lack the precision of advanced CFD solvers and are not designed for highly accurate biomechanics or particle-fluid interactions.

Future research directions may include:

1. Using high-precision eye movement recording technology to analyze nystagmus characteristics in detail during each step of the Epley and Semont maneuvers.
2. Developing more precise virtual simulation models that include more anatomical details and individual difference factors.

## 5 Conclusion

Through virtual simulation technology and literature review, we have gained a deeper understanding of the mechanisms and clinical significance of the Tumarkin-like phenomenon in the final step of the Epley and Semont maneuvers. Specifically, a correctly performed Epley maneuver should not evoke nystagmus in its final step. However, for the Epley maneuver, sitting-up vertigo was often linked to treatment failure because it was seen as a sign that the canaliths had not been successfully repositioned or that there was ongoing irritation within the semicircular canals. But if the head rotation angle in Step D is <120° from the horizontal position, the Tumarkin-like phenomenon may indicate success. In contrast, for the Semont maneuver, sitting-up vertigo was often linked to successful treatment. However, when the head is maintained at or above the horizontal plane during the opposite lateral decubitus maneuver, the otoliths are able to directly enter the utricle via the common crus, and in such cases, sitting-up vertigo may be linked to treatment failure. These findings not only help explain clinically observed phenomena but also provide new perspectives for optimizing BPPV treatment strategies. In the future, more clinical studies are needed to verify these virtual simulation results, thereby developing more precise and personalized BPPV diagnosis and treatment methods.

## Data Availability

The original contributions presented in the study are included in the article/Supplementary material, further inquiries can be directed to the corresponding author.

## Appendix

### 1. Epley maneuver, final step without nose-down position

1. Animation A1.1. Observation of otolith movement on the long arm side of the left posterior semicircular canal (Supplementary video S1).
2. Animation A1.2 Observation of otolith movement on the short arm side of the left posterior semicircular canal (Supplementary video S2).
3. Animation A1.3 Observation of otolith movement within the left utricle (Supplementary video S3).

### 2. Epley maneuver, step D positions

1. Animation A2.1. Lateral decubitus position (Supplementary video S4).
2. Animation A2.2. Semi-prone position (Supplementary video S5).
3. Animation A2.3. Prone position (Supplementary video S6).

### 3. Epley maneuver, final step with 45° nose-down position

1. Animation A3.1. Observation of otolith movement on the long arm side of the left posterior semicircular canal (Supplementary video S7).
2. Animation A3.2. Multi-position otolith movement observation in the left posterior semicircular canal (Supplementary video S8).

### 4. Semont maneuver positions

1. Animation A4.1. Step B: lateral decubitus position above the horizontal plane (Supplementary video S9).
2. Animation A4.2. Step C: opposite lateral decubitus with head at the horizontal plane (Supplementary video S10).
3. Animation A4.3. Step C: opposite lateral decubitus with head below the horizontal plane (Supplementary video S11).
